# Integration and Outcomes of Entrustable Professional Activities Performed by PharmD Trainees at Hospital Practice Sites

**DOI:** 10.3390/pharmacy14050115

**Published:** 2026-08-06

**Authors:** Tania Bayoud, Sarah Alghanem, Fajer Al-Sejari, Mai Alhazami, Pierre Moreau

**Affiliations:** College of Pharmacy, Health Sciences Centre, Kuwait University, P.O. Box 24923, Safat 13110, Kuwait; sara.alghanem@ku.edu.kw (S.A.); alsejarif@outlook.com (F.A.-S.); mai.alhazami@ku.edu.kw (M.A.); pmoreauconsulting@outlook.com (P.M.)

**Keywords:** drug therapy problems, entrustable professional activities, hospital, ambulatory care, medication therapy management, medication reconciliation, PharmD trainees, competency-based education

## Abstract

Objectives: Our primary objectives were to evaluate the integration and impact of Entrustable Professional Activities (EPAs) within a competency-based add-on PharmD curriculum and to describe the types and frequency of EPAs performed by PharmD trainees as an assessment tool during their placements at secondary and tertiary care practice sites. A secondary objective was to report, categorize, and quantify patients’ drug therapy problems (DTPs) with the rate of recommendations’ acceptance by preceptors. Methods: A descriptive study for 2437 EPAs (concerning 1295 patients) was performed by 56 PharmD trainees during their experiential training year from January 2018 to November 2021. Results: The most frequent EPA performed by trainees was Medication Therapy Management (MTM) (718; 29.5%), followed by Medication Reconciliation (MedRec) (636; 26.1%), prescription/order review (546; 22.4%), and medication counseling (284; 11.7%). A total of 1224 DTPs were encountered through MTM, MedRec, and prescription/order reviews, with “drug not indicated” being the most common DTP (16.7%). Anti-infectives (25.1%), cardiovascular (21.8%), endocrine (12.5%), and gastrointestinal (12.1%) agents made up most of the 1612 medicines involved in medication-related DTPs. The majority of interventions were accepted by preceptors (91%). The rejection rate was 3.6%, owing primarily to medical expertise, clinical judgment, and the personal opinion of preceptors. Patients’ age (older adults), gender (females), and admission type (newly admitted patients) were associated with a higher risk of experiencing DTPs. Conclusions: Our findings demonstrate that incorporating EPA-based practice and assessment into pharmacy training can ensure graduates are not only knowledgeable but also trusted to perform essential tasks independently, ultimately optimizing the safety and quality of patient care.

## 1. Introduction

Health care is becoming more complex, creating a need to graduate healthcare providers equipped with adequate knowledge and capable of managing complex problems. This drives a shift in healthcare education to adopt a competency-based education (CBE) approach. Several competency frameworks have been developed for medicine, nursing and pharmacy [1,2,3]. Competency practice and assessment can be operationalized and assessed by linking them with professional activities [4,5]. In light of this, the College of Pharmacy at Kuwait University implemented a two-year post-baccalaureate add-on PharmD program from 2016 to 2025 to graduate competent pharmacists in providing more clinically oriented services. The program is competency-based, and professional activities are tailored to the population and country needs [6,7]. The curriculum is built around a locally developed framework of Entrustable Professional Activities (EPAs) that represent the essential professional tasks expected of graduates. The framework was developed through a national needs assessment involving multiple stakeholders focus groups, including pharmacists from different practice settings, physicians, patients, educators, and healthcare leaders, followed by iterative faculty refinement and alignment with international competency frameworks, including those of the International Pharmaceutical Federation (FIP), Centre for the Advancement of Pharmacy Education (CAPE), National Association of Pharmacy Regulatory Authorities (NAPRA), and the Pharmaceutical Society of Ireland. This process resulted in a pharmacy-specific EPA framework tailored to Kuwait’s healthcare needs, encompassing activities such as medication therapy management (MTM), medication reconciliation (MedRec), patient counseling, dispensing, extemporaneous preparation (EP), therapeutic drug monitoring (TDM), health promotion, and medication information services. The EPAs form the backbone of the curriculum by integrating knowledge, skills, and professional behaviors into clinical tasks that are introduced in simulated learning environments before being progressively performed and assessed during experiential training under preceptor supervision. The complete competency framework and EPA description, including its development process and associated competency profiles, have been published in detail elsewhere [6] and have not been repeated in this manuscript. The focus of the manuscript is the use of these EPAs during the experiential learning phase of the program.

Within the curriculum, EPAs are first introduced and practiced on campus in the form of case-based laboratories and roleplay scenarios, whereas the second year focuses on clinical rotations with hands-on practice using a subset of EPAs at different placement sites. EPAs have a holistic nature; they require several competency domains that integrate adequate knowledge, skills and attitudes that are acquired through education. Hence, as students progress, they are gradually entitled or trusted to perform EPAs with increasing autonomy [8]. In pharmacy education, EPAs have been increasingly embraced to guide competency-based curricula, particularly during experiential training, by aligning educational outcomes with hands-on clinical practice [9]. Previous studies have demonstrated that EPA-based assessment provides a structured framework for workplace-based assessment, enabling preceptors to directly observe students performing patient care activities, provide timely formative feedback, and make informed entrustment decisions regarding students’ readiness for independent practice [6]. Compared with traditional assessment approaches that often evaluate knowledge or isolated competencies, EPAs integrate multiple competencies within patient care activities, thereby enabling their assessment while supporting progressive entrustment and learner development. A scoping review found that a major motivation for EPA use in entry-level health professional education is to improve patient safety by aligning performance and expectations and to improve student assessment [10]. EPAs can help supervisors in their determination of trainees’ competence and personalization of feedback [11]. Despite the increasing adoption of EPAs in competency-based pharmacy education, there is limited evidence describing their implementation and outcomes in PharmD experiential training, particularly within Kuwait and the Gulf region.

In a survey performed back in 2018 that assessed pharmacy practices in Kuwait, the most common services documented and carried out by clinical pharmacists were patient education and drug information [12]. The study identified barriers affecting the implementation of pharmacy clinical services, including lack of policy, time, and clinical skills [12]. One retrospective cross-sectional study conducted in a private hospital in Kuwait supported the clinical practice of pharmacists by highlighting their positive impact on patient care through the identification and resolution of drug therapy problems (DTPs) [13]. Similarly, another national retrospective observational study found that the best possible medication history (BPMH) completed by pharmacists was a strategy to reduce polypharmacy, notably the use of over-the-counter medications. Studies have shown that pharmacist-led BPMH contribute to reducing hospital readmissions and healthcare costs, while improving patient outcomes and quality of life [14,15,16]. Roles played by students and trainees are of equal importance to that of licensed pharmacists, empowering them as great assets in the health care system [17,18]. Despite the fact that their primary role is educational and strictly performed under supervision, focusing on learning the principles of clinical practice, pharmacology, and patient communication, several studies highlight their prominent influence on the health care system [19,20,21,22,23,24,25,26]. A study by Gortney and colleagues found that student-obtained medication histories helped identify DTPs and improve patients’ outcomes in terms of reducing emergency department visits within 30 days post discharge [27]. Clinical pharmacy trainees were found to be a promising solution for workforce limitations, whether related to tight time or number of clinical pharmacists, during medication reconciliation activities for patients at transitions of care in Australia [28]. A study by Phelps and colleagues showed the positive impact of medication reconciliation performed by clinical trainees on discharged patients at high risk of admission on reducing readmission rates [29]. Even in pediatric practice that requires more specialized competencies, pharmacy students proved their positive effect on practice and cost savings [30]. The positive influence of incorporating students in practice goes beyond the health care system, as evidence has shown that students improve their confidence and gain a better understanding of interprofessional care [31]. A survey conducted on students participating in the transitions-of-care revealed the positive impact of their active participation on self-perceived growth of autonomy [32]. Also, early implementation of students in clinical activities and early training in information documentation were shown to increase students’ confidence [33]. Such findings encourage a collaborative dynamic between clinical pharmacists and trainees to foster a work-based learning environment that enhances the trainees’ competencies while maintaining high standards of care.

The objective of this study is to evaluate the integration of EPAs within a competency-based PharmD curriculum by (1) identifying the types of EPAs performed by PharmD trainees during their placements under preceptors’ supervision at secondary and tertiary care practice sites in Kuwait, (2) categorizing and quantifying encountered DTPs along with the medication classes most frequently involved, and (3) assessing the rate and reason of recommendations’ acceptance by preceptors.

## 2. Methods

### 2.1. Study Design, Setting, and Population

A retrospective descriptive study was conducted from January 2018 to November 2021, during their experiential training year at public hospitals in Kuwait for both secondary and tertiary care. The PharmD trainees were over 21 years old, had finished a 5-year bachelor’s degree, and were in their second year of an Add-on PharmD program. Experiential training encompassed a variety of clinical specialties including departments of intensive care units (ICU), pediatric ICU, infectious disease, geriatrics, gastroenterology, nephrology, and surgery. A gap period, from 2020 to 2021, during the COVID-19 pandemic, took place as students were not allowed to perform their curriculum-based activities on site in compliance with governmental laws and restrictions for their own safety.

During the second year of the PharmD program, students complete six experiential rotations, each lasting six weeks. The first semester entails three rotations: (1) a general medicine hospital rotation, during which students practice four EPAs, including MedRec, MTM, dispensing, and EP; (2) an ambulatory care rotation in a polyclinic setting, where students perform four EPAs, including MedRec, patient counseling, dispensing, and health promotion activities; and (3) a Non-Direct Patient Care (NDPC) practice experience. The NDPC rotation allows students to participate in a variety of pharmacy-related activities and projects aimed at developing complementary competencies and broadening their understanding of the diverse roles and services within the pharmacy profession. During the second semester, students complete three hospital-based specialty rotations selected according to their interests from a range of specialties, including intensive care, cardiology, pediatrics, geriatrics, infectious diseases, nephrology, psychiatry, surgery, gastroenterology, and oncology, where students perform MedRec, MTM, EP, patient counseling, and TDM.

Each EPA is linked to a designated Competency Demonstration Activity (CDA) profile that outlines the specific competency elements to be assessed. Student performance is evaluated based on the level of entrustment granted by the preceptor, reflecting the degree of supervision and guidance required while performing the activity: level 1—do not trust that the trainee can perform (observation only); level 2—trust that the trainee can perform under continuous guidance; level 3—trust that the trainee can perform with moderate guidance (re-active supervision); and level 4—trust that the trainee can perform without guidance (or supervision). Students are expected to perform at levels 1–2 during the first week of each rotation and progressively advance toward level 4 for all competency elements by the end of the rotation. EPAs are assessed formatively on a weekly basis to provide continuous feedback, while global assessments evaluating broader competencies, such as professionalism and lifelong learning, are conducted at the midpoint and end of each rotation. Summative EPA assessments are completed at the conclusion of each rotation and submitted to the faculty experiential coordinator. In addition, students are required to submit EPA-specific deliverables, which are evaluated by assigned faculty members and contribute to the final course grade alongside the summative EPA and global assessment scores. Each competency element demonstrated at the expected level (e.g., level 4) receives a mark of 1, one level below expectation (e.g., 3 when 4 is expected) receives 0.5, and two levels below expectations (e.g., 2 when 4 is expected) receives zero (0). Marks for all competency elements are then added to give the score for the activity. For example, an EPA comprising 15 competency elements has a maximum score of 15, which is subsequently converted into the percentage allocated to that EPA within the rotation.

Throughout the experiential program, students are supervised and evaluated by trained preceptors. Rotation sites and preceptors are selected according to predefined criteria to ensure that all required EPAs can be appropriately practiced and assessed. Prior to supervising students, all preceptors must successfully complete the institution’s Preceptor Training Program, which consists of two modules: General Preceptor Skills (GPS) and Assessment of Pharmacy Students (APS). Comprehensive rotation manuals are provided to both students and preceptors and contain all information relevant to the experiential program, learning objectives, assessment procedures, and rotation requirements.

For each EPA, student recommendations and clinical interventions are documented and evaluated. Acceptance of these recommendations is determined based on their evidence-based appropriateness and clinical correctness as assessed by the supervising preceptor during the experiential rotation.

### 2.2. Ethical Approval

Ethical approval was obtained from the Kuwaiti Ministry of Health and the Health Science Centre Ethical Committees (REC/no 1263/2022) at Kuwait University on 31 May 2022. Owing to the retrospective nature of the study, the need for informed consent was not mandatory.

### 2.3. Data Collection Tool/Form

EPA forms were completed and collected by PharmD trainees in a paper form and stored at the College of Pharmacy, Kuwait University, since January 2018. Each form was divided into five sections, including patient demographics and clinical presentation; EPA types [medication counseling, MedRec, Medication Therapy Management (MTM), therapeutic drug monitoring (TDM), and others]; drug therapy problems; description of intervention with the drug class involved; and preceptor response. It must be noted that MedRec activities included conducting BPMH and reconciling encountered discrepancies. Forms completed by the trainees throughout their rotations in different hospital specialties and sites were included in this study. It must be noted that the classification of DTPs encountered in this study was adapted from Strand et al. with modifications [34].

### 2.4. Statistical Analysis

Data were transcribed to Microsoft Forms. Descriptive analysis was conducted in Microsoft Excel. Data were presented as frequencies and percentages.

Categorical variables, including age group, gender, and admission type were analyzed for associations with DTPs using the Chi-Square (χ^2^) test of independence. Statistical significance was set at *p* < 0.05. All statistical analyses were performed using Python 3.14.3 (Pandas 3.0.5 and SciPy 1.18.0 libraries).

## 3. Results

A total of 1295 EPA forms were analyzed, each form representing one patient, and a total of 2437 EPA types were performed (one form can have >1 EPA type). Activity forms were primarily documented during the ICU rotation (416 patients; 32.1%), followed by nephrology (246 patients; 19%), general medicine (209 patients; 16.1%), and pediatric ICU rotation (202 patients; 15.6%). Forms were completed mainly for adults with a median age of 56 years (IQR (41–73)). Approximately 47% were enrolled as new patients, most of whom had cardiovascular (CV) disease (46.9%), with hypertension (24.6%) and diabetes (20.9%) being the predominant diseases. The detailed patients’ demographics and clinical characteristics are summarized in Table 1.

Out of a total of 2437 activities, MTM was the most commonly performed by the trainees (n = 718; 29.5%). MedRec (636; 26.8%) came in second, followed by prescription/order review (546; 23%), medication counseling (284; 12%), and drug information (166; 7%). Table 2 summarizes all activities carried out by PharmD trainees at their sites. It must be noted that before commencing their advanced pharmacy practice experiences, students practiced all EPAs during the Advanced Pharmacotherapy course using the same CDA profile and entrustment framework applied during clinical rotations. At this stage, students performed the EPAs in a simulated classroom environment under close instructor supervision, with competency determined by the level of guidance required. This formative phase provided a structured opportunity for students to develop their skills, learn from mistakes, and receive continuous feedback before entering clinical practice. During the second year of the PharmD program, students progressively applied the same EPAs across their hospital rotations with increasing levels of independence. As competency developed through repeated practice and preceptor assessment, students were expected to achieve Level 4 entrustment, reflecting the ability to perform the activity independently with no guidance or supervision by the end of their rotations. The attained entrustment level contributed to the overall assessment of student performance and final rotation grade, reflecting their progression toward independent clinical practice.

A number of DTPs were identified while conducting different EPAs (mainly MTM, MedRec, and prescription/order review), resulting in a total of 1224 issues (average of two DTPs per patient). “Drug not indicated” was the most frequently encountered DTP in practice (16.7%). Clinical trainees also adjusted several medication dosages because, depending on the indication, certain doses were either too high (14.3%) or too low (13.8%). On the other hand, some patients’ conditions were found to be untreated (8.8%), and the trainee addressed this issue by recommending the appropriate medication. Numerous additional DTPs that required interventions were managed (see Table 3).

The preceptor’s acceptance rate of trainee recommendations was remarkably high (91% overall). Around 3.6% of the interventions were not accepted, and the most common reasons were preceptor expertise, clinical judgment, and personal clinical opinion. The detailed DTPs identified along with the acceptance rate and involved drug classes are listed in Table 3.

Of a total of 1612 drugs, anti-infectives accounted for the highest percentage of medications involved in interventions (25.1%). Other medications involved included cardiovascular (21.8%), endocrine (12.5%), and gastrointestinal (GI) (12.1%) agents. Many patients were given proton pump inhibitors, which comprised the bulk of GI agents.

The statistical analysis revealed significant relationships between key demographic factors and the occurrence of DTPs. The Chi-Square (χ^2^) test indicated that age, gender, and admission type were all strongly associated with the likelihood of experiencing DTPs, with *p*-values well below 0.001 (refer to Table 4), confirming statistical significance. Notably, older adults, females, and newly admitted patients were more likely to experience medication-related issues.

## 4. Discussion

To our knowledge, this is the first study in Kuwait to provide a comprehensive description of PharmD trainees’ EPAs outcomes across multiple hospital sites and specialties, underscoring the vital role of CBE in advancing pharmacy practice within the national healthcare system. The competency framework and EPA matrix developed by Moreau et al. and Al-Taweel et al. [6,7] have helped the Add-on PharmD program align training with both local healthcare needs and international standards. Building on this foundation, EPAs were practiced in a controlled in-class environment during the first year of the add-on PharmD to prepare PharmD trainees to effectively apply their competencies in real-world practice settings during their clinical rotations of the second year.

Conducted on a large, multi-year dataset, the study captures the diversity of services provided and examines the types of EPAs performed by PharmD trainees during their clinical placements as a way of assessing real-world competencies. EPAs offer a structured framework for translating clinical skills into observable, measurable tasks, making them a valuable tool for both training and professional assessment [8].

Based on the results above, the most frequently performed EPA by the trainees was MTM, reflecting its central role in direct patient care. This was followed by MedRec and prescription/order review. These findings contrast with earlier reports from Kuwait, where patient education and drug information were the predominant services [12], reflecting a curriculum-driven shift toward more complex, patient-centered clinical activities. This demonstrates the CBE principle of progressive entrustment, where trainees gradually advance from needing supervision to becoming autonomous in completing more complex clinical tasks. The year of on-campus training with the same EPAs has supported students in developing a reasonable degree of autonomy by the time they began their rotations. The rising prominence of MedRec, which was once one of the least provided services in Kuwait [12], mirrors international evidence of its value in reducing medication discrepancies and enhancing patients’ safety [28,29]. For example, a retrospective study by Alghanem et al. in Kuwaiti dialysis patients showed that pharmacist-led best possible medication histories (BPMH) helped reduce polypharmacy and uncovered unnecessary drugs (often over-the-counter), consistent with our DTP results [35]. Additionally, patient education, which is often reported as the leading activity performed by PharmD students in other studies [13,14,27,30,36,37], was also evident in the present study, yet as a supporting rather than a dominant professional activity. Taken together, these results suggest that pharmacy training in Kuwait has shifted from primarily providing information and counseling, toward delivering more collaborative, clinically oriented services that focus on optimizing therapy decisions and improving patient outcomes.

While performing EPAs, trainees encountered several DTPs, often multiple ones for the same patient. With respect to the type, “Drug not indicated” was the most encountered, which refers to a drug being used without an apparent medical indication, highlighting the issue of polypharmacy in prescribing practice in Kuwait. Our finding was in contrast with other similar global studies, where drug omission (as in “indication not treated”) was the leading DTP encountered [20,21,22,23,24,26,28]. From a CBE perspective, this highlights the importance of integrating evidence-based decision-making competencies into EPA design.

Similar to global findings, the class of anti-infective medications was the most involved in DTPs [11,28,29]. This supports the need for pharmacist-led antimicrobial stewardship initiatives to resolve the current practice gap. Wanishayakorn et al. found in their work that MedRec and dose adjustments concerning antibiotics were the major source for cost savings [38]. Interestingly, a study by Bio and colleagues assessed the factors influencing pharmacy students’ self-initiation of interventions during patient encounters in acute patient care and identified that fewer encounters were associated with infectious disease cases [39]. The authors justified their finding to the inherent difficulty of identifying the exact causative organism and the complexity of antibiotics as a challenging topic for students. However, this was not observed among the students in this study, reflecting strong knowledge of infectious disease treatment and effective application in practice, likely the consequence of a robust competency-based educational approach before the rotations.

GI drugs still appear to be among the most implicated drugs in DTPs, mirroring the results of Bayoud et al. [13]. We noticed significant shifts in the rankings with the emergence of CV agents, endocrine and metabolic agents, and even central nervous system (CNS) agents from the fourth, sixth, and ninth places as reported by Bayoud et al. to the second, third, and sixth places, respectively [13]. In fifth place, hematological products, specifically thromboprophylaxis agents, were mentioned, suggesting that physicians experienced difficulties dealing with these drugs and the presence of a clinical pharmacist is essential for efficient and safe care. It is important to note that our study was conducted in public hospitals, whereas the other study was conducted in a private hospital setting. Differences in patient populations, case complexity, and prescribing patterns between public and private hospitals may justify the variations observed in the ranking of used drug classes. Public hospitals in Kuwait typically manage a higher number of acutely ill, multimorbid, or high-risk patients, which may contribute to the increased involvement of cardiovascular, endocrine, CNS, and hematology agents in the DTPs reported in this study.

The high acceptance rate for student recommendations by their preceptors in this study (91%) was in accordance with other studies that also reported high (>80%) acceptance rates of pharmacy trainees’ recommendations by preceptors [19,22,24,26,33]. Our observations exceeded some earlier data obtained in Kuwait [12,13]. Such a high current acceptance rate shows that trainees have reached a level of competence and trust, whereby their clinical recommendations are recognized by experienced practitioners as safe, appropriate, and genuinely valuable.

Elderly patients were found to be at higher risk of DTPs, aligning with global research that highlights the increased risk of polypharmacy, adverse drug reactions (ADRs), and inappropriate prescribing in elderly populations [40,41]. Similarly, gender differences played a significant role, as seen in studies from Europe and North America, where women have been reported to experience a higher incidence of medication-related complications compared to men [42,43]. In addition, the current data revealed that newly admitted patients were at greater risk of presenting DTPs compared to follow-up cases, potentially reflecting multiple and disconnected initial diagnoses and the need for medication adjustments upon admission. This finding is consistent with international reports where medication reconciliation at the point of hospital admission is a critical intervention to reduce prescribing errors [44]. Within our CBE approach, structured exposure to these patient groups (elderly, females, newly admitted) through targeted EPA assignments can strengthen trainees’ abilities to handle complex, high-risk cases with confidence, ultimately improving patients’ outcomes.

It must be noted that exposing pharmacy students to not only different specialty rotations but also different aspects of pharmacy during their academic years, such as community pharmacies and the industry sector, helps them better decide their preference and future careers. A study by Dupuis et al. reported that hospital experiential rotations and interactions with preceptors significantly influenced fourth-year pharmacy students’ decisions at the Faculty of Pharmacy-Université de Montréal to pursue hospital pharmacy residencies [45]. These experiences led some students to change their career orientation during their final year [45]. Another study in Saudi Arabia investigated the factors influencing future career interests of pharmacy interns [46]. Internship training was found to be strongly associated with the pursuit of a clinical pharmacy career, while financial incentives often guided students toward roles in the pharmaceutical industry. Additionally, exposure to academic content through college coursework influenced some students to consider careers in academia, whereas summer training programs were more closely linked to the choice of community pharmacy practice [46]. Compared to other Gulf Cooperation Council (GCC) countries, Kuwait’s clinical pharmacy practice is still evolving and gaining momentum. Saudi Arabia, the United Arab Emirates, and Qatar have expanded pharmacists’ roles through collaborative drug therapy management, chronic disease care, and immunization services, supported by strong academic and institutional frameworks [47,48,49,50]. This study documents Kuwait’s progress toward a more advanced clinical pharmacy practice.

A key strength of this study is the large sample size and the number of documented EPAs collected over a period of four years. This extensive dataset provides a comprehensive overview of PharmD trainees’ activities across diverse hospital specialties and allows for a robust assessment of competency development in real-world practice. The multi-year design also reflects the variability in service delivery, enhances the reliability of observed patterns, and offers a stronger foundation for generalizability across Kuwait’s secondary and tertiary care settings. Additionally, the study involves multiple rotation sites, which strengthens the representativeness of the findings and reflects the diversity of clinical environments in which trainees practice. It is important to note that this work represents the final outcome of the framework introduced by Moreau et al. [6], translating it from a classroom-based concept into a practical, hands-on approach applied in real-life settings.

Despite these strengths, certain limitations must be acknowledged. The study did not correlate EPAs with clinical outcomes, patient-reported measures, or healthcare cost-effectiveness data, which limits the ability to quantify the long-term impact or economic value of trainees’ contributions. Additionally, the retrospective design relies on the accuracy and completeness of trainee-documented EPA forms, which may introduce inconsistencies in categorization or reporting of activities and DTPs. To address this issue, a group of three academic preceptors met to review and discuss the cases, subsequently reaching a consensus on standardized definitions and providing clarification and guidance to the trainees. Notably, this study exposed students’ documentation issues that will be addressed in the future for improvement. It is vital to have a uniform, standardized worksheet to guide student documentation and, later, enable better evaluation of findings [20,51].

## 5. Conclusions and Future Directions

This study demonstrates that embedding entrustable professional activities (EPAs) within a competency-based curriculum (CBE) can successfully prepare healthcare graduates for clinically relevant, patient-centered care across multiple specialties. The results showed that trainees delivered a wide range of EPAs, particularly medication therapy management (MTM), medication reconciliation (MedRec), and prescription/order reviews, reflecting a deliberate move toward more complex, integrative services with a high acceptance rate. The results confirm that EPA-based training nurtured competence and professional assurance. By linking classroom learning with real-world practice, this research highlights that EPAs assist in building the competence required for independent pharmacy practice and strengthen the role of trainees in enhancing patient safety and evolving healthcare demands and expectations. Targeted EPA assignments focusing on vulnerable populations, particularly older adults, females, and newly admitted patients, could further advance medication safety and bring practice in line with global safety standards. Future research should assess the long-term impact of EPA-driven training on clinical effectiveness, quality of life, patient satisfaction, and healthcare costs. Demonstrating the cost-effectiveness of pharmacist-led activities may also influence policy and optimize resource allocation, reinforcing the role of PharmD graduates as integral members of healthcare teams.

## Figures and Tables

**Table 1:** Demographics, including age, gender, specialty, type of admission, and comorbidities, of the patients for whom an EPA was performed.

	Characteristics	(n)	Frequency (%)
Age (years)	- 0–12 - 13–17 - 18–29 - 30–44 - 45–59 - 60–74 - >75 - Missing data - Median	- 183 - 18 - 48 - 166 - 305 - 366 - 160 - 49 - 56	- 14.1% - 1.4% - 3.7% - 12.8% - 23.5% - 28.3% - 12.4% - 3.8%
Gender	- Male - Female - Missing data	- 675 - 571 - 49	- 52.1% - 44.1% - 3.8%
Specialty	- Adult ICU - Nephrology - Medicine - Pediatric ICU - Infectious disease - Gastroenterology - Surgery - Geriatrics - Missing data	- 416 - 246 - 209 - 202 - 103 - 53 - 29 - 26 - 11	- 32.1% - 19.0% - 16.1% - 15.6% - 8.0% - 4.1% - 2.2% - 2.1% - 0.8%
Type of admission	- New patient - Follow-up - Other (transferred, post-op, receiving fluids) - Missing data	- 611 - 339 - 98 - 247	- 47% - 26% - 8% - 19%
Comorbidities	Cardiovascular disorders - Hypertension - Coronary artery disease - Dyslipidemia - Stroke/Transient ischemic attack - Atrial fibrillation - Heart failure - Deep vein thrombosis - Others Endocrinologic disorders - Diabetes - Thyroid/parathyroid disorders - Others Renal disorders - Chronic kidney disease/End stage renal disease - Electrolyte imbalance Respiratory disorders - Asthma - Chronic obstructive pulmonary disease Neurological disorders Rheumatologic disorders Gastrointestinal disorders Psychiatric disorders Hematologic disorders Urologic disorders Oncologic disorders Immunologic disorders Ophthalmic/Otolaryngological disorders Others (genetic disorders, amputation, transplant rejection, vitamin deficiency, malnutrition, substance abuse) Missing	1401 - 752 - 328 - 119 - 90 - 55 - 28 - 7 - 22 724 - 636 - 85 - 3 241 - 234 - 7 159 - 130 - 29 138 79 79 54 38 24 18 16 5 69 3	46.9% - 24.6% - 10.8% - 3.9% - 3.1% - 1.8% - 1.0% - 0.3% - 0.7% 23.8% - 20.9% - 2.8% - 0.1% 7.9% - 7.7% - 0.2% 5.4% - 5.2% - 4.3% 4.5% 2.6% 2.6% 1.8% 1.2% 0.8% 0.6% 0.3% 0.2% 2.3% 0.1%

**Table 2:** Entrustable professional activities performed by clinical trainees during the study period (from the most performed to the least).

Entrustable Professional Activity	Frequency	Percentage
Medication therapy management (MTM)	718	29.5
Medication reconciliation (MedRec)	636	26.1
Best possible medication history (BPMH)	394	16.2
Discrepancy reconciliation	242	9.9
Prescription/Order review	546	22.4
Medication counseling	284	11.7
Drug information	184	7.5
Therapeutic drug monitoring (TDM)	69	2.8
Total	2437	

**Table 3:** Drug therapy problems, acceptance rates, and drug classes during the study period.

	Characteristics	(n)	Frequency (%)
Drug Therapy Problems	- Drug not indicated - Subtherapeutic dose - Untreated indication - Supratherapeutic dose - Optimize frequency - ADR/Unsafe for patient - Drug interaction - Ineffective Drug-Need to change - Therapeutic duplication - Non-adherence - Optimize dosage form - Monitoring (labs and vital signs) Total	- 205 - 175 - 108 - 169 - 126 - 131 - 105 - 52 - 47 - 44 - 27 - 35 1224	- 16.7% - 14.3% - 8.8% - 13.8% - 10.3% - 10.7% - 8.6% - 4.3% - 3.8% - 3.6% - 2.3% - 2.8% 100%
Preceptor Acceptance	- Accepted - Not accepted - No response - Modified - Others (patient left before response, agreed but not applied, discussed with patient, patient passed away)	- 2062 - 81 - 50 - 60 - 14	- 91% - 3.6% - 2.2% - 2.6% - 0.6%
Drugs Classes Included	- Anti-infectives (anti-bacterial, anti-viral, and anti-fungal agents) - CV agents (ß-blockers, diuretics, statins, fibrates, and anti-platelets) - Endocrine and metabolic disease agents (incretin mimetics, insulins, and thyroid hormones) - GI agents - Hematology/Thromboprophylaxis agents - CNS agents - Respiratory agents - OTC agents - Allergy/Immune system agents - Analgesics and antipyretics - Electrolytes Total	- 405 - 351 - 201 - 195 - 124 - 104 - 71 - 44 - 38 - 32 - 47 1612	- 25.1% - 21.8% - 12.5% - 12.1% - 7.7% - 6.5% - 4.4% - 2.7% - 2.4% - 2% - 2.9% 100%

**Table 4:** Results of statistical analysis of current dataset vs. Drug Therapy Problems (1224).

	Chi-Square (χ^2^)	*p*-Value
Age Group		
Elderly >60 yrs = 526 (40.6%)	997.1	<0.001
Gender		
Female = 571 (44.1%)	2024.6	<0.001
Admission Type		
New patients = 611 (47%)	5848.5	<0.001

## Data Availability

The original contributions presented in this study are included in the article. Further inquiries can be directed to the corresponding author.
